# Genetic Variability of *Arabidopsis thaliana* Mature Root System Architecture and Genome-Wide Association Study

**DOI:** 10.3389/fpls.2021.814110

**Published:** 2022-01-28

**Authors:** Agnieszka Deja-Muylle, Davy Opdenacker, Boris Parizot, Hans Motte, Guillaume Lobet, Veronique Storme, Pieter Clauw, Maria Njo, Tom Beeckman

**Affiliations:** ^1^Department of Plant Biotechnology and Bioinformatics, Ghent University, Ghent, Belgium; ^2^VIB Center for Plant Systems Biology, Ghent, Belgium; ^3^Forschungszentrum Jülich GmbH, Agrosphere (IBG-3), Jülich, Germany; ^4^Gregor Mendel Institute of Molecular Plant Biology, Vienna, Austria

**Keywords:** root systems architecture, genome-wide association study, *Arabidopsis thaliana*, ecotypes, accession, root development, rhizotron

## Abstract

Root system architecture (RSA) has a direct influence on the efficiency of nutrient uptake and plant growth, but the genetics of RSA are often studied only at the seedling stage. To get an insight into the genetic blueprint of a more mature RSA, we exploited natural variation and performed a detailed *in vitro* study of 241 *Arabidopsis thaliana* accessions using large petri dishes. A comprehensive analysis of 17 RSA traits showed high variability among the different accessions, unveiling correlations between traits and conditions of the natural habitat of the plants. A sub-selection of these accessions was grown in water-limiting conditions in a rhizotron set-up, which revealed that especially the spatial distribution showed a high consistency between *in vitro* and *ex vitro* conditions, while in particular, a large root area in the lower zone favored drought tolerance. The collected RSA phenotype data were used to perform genome-wide association studies (GWAS), which stands out from the previous studies by its exhaustive measurements of RSA traits on more mature *Arabidopsis* accessions used for GWAS. As a result, we found not only several genes involved in the lateral root (LR) development or auxin signaling pathways to be associated with RSA traits but also new candidate genes that are potentially involved in the adaptation to the natural habitats.

## Introduction

Crops are in high need of improvement for higher yield and resistance to environmental stresses (Ray et al., 2013). The focus on the yield, however, makes it easy to forget that one of the most important parts of the plant cannot be seen: the hidden underground. Due to ongoing climate changes, all over the world, the breeders look for ways of modulating underground plant parts to improve the overall plant growth and yield efficiency (Rogers and Benfey, 2015; Chen et al., 2019). To obtain this goal, they focus on root system architecture (RSA) which is a concept used to summarize and calculate all aspects of root development. The RSA describes the spatial configuration of the overall root structure in the substrate it is growing in and includes the initiation and development of different subparts of the root and the speed of root growth. The RSA adapts to a changing environment and is responsible for the most optimal nutrient uptake depending on the conditions (Oldroyd and Leyser, 2020). *Arabidopsis thaliana* has been used as a model organism to study all aspects of plant biology and the root has turned out to be a very elegant model organ for cell and developmental biology studies. Typically, roots are studied on young seedlings grown for a short period on vertical agar plates, commonly for 5 or 7 days (Zhao et al., 2017; Ogura et al., 2019), a developmental phase characterized by a still premature root system hardly showing higher order LRs. To our knowledge, up to date, only one study analyzed traits 16 days after sowing, but in this case, the roots were grown under different stresses which had a negative influence on their overall size (Rosas et al., 2013).

It is very well known that RSA can strongly vary depending on the environmental conditions that influence the three-dimensional distribution of the primary and LRs (Del Bianco and Kepinski, 2018). But plasticity of traits is also driven by genetic variation that shapes RSA. Within one species, local populations might have adapted their RSA to the prevailing environmental conditions which might have become a heritable trait. Such root types have obtained a genetic imprint and could be used to get insight into the genetic basis for root system adaptation to environmental conditions. A previous study demonstrated the wide natural variability in RSA among 12 *Arabidopsis* accessions that were collected originally from different geographical areas (Aceves-García et al., 2016).

In this study, we analyzed the RSA of 241 accessions incubated for 21 days in large Nunc plates (24 cm × 24 cm). The results show a broad range of variability for all parameters tested and represent a unique description of the existing natural variation in root system architecture in the *Arabidopsis* species. A selection of four accessions with a contrasting RSA was tested in rhizotrons and subjected to water limiting conditions to correlate root traits with plant performance in drought conditions. Finally, a GWA study was conducted, indicating potential genetic loci responsible for the implementation of specific root system architectures.

## Materials and Methods

### Plant Material

A seed collection of 328 *A. thaliana* accessions has been selected from the Hap-Map population based on habitat, geographical information, or previously described interesting root or/and shoot phenotype. About 120 accessions were obtained from an in-house collection derived from the Nottingham Arabidopsis Stock Centre (NASC), the rest was retrieved directly from NASC (Scholl et al., 2000). The lines that did not germinate, flower, or perform poorly, were discarded from the analysis. The final set of plant material, that has been subjected to root analysis included 241 accessions (Supplementary Table 1). Seeds were gas-sterilized with chlorine (150 ml NaOCl with 8 ml HCl, overnight, in a closed container). Next, the seeds were vernalized at 4°C for 4 days. The values of climate temperature and precipitation have been collected for precise longitude and latitude locations, for the years: 1989–2019 from an online climate data source^1^. Soil types have also been noted based on the geographical localization of accessions, sourced from an online data source^2^.

### Growth Conditions and Image Acquisition

Phenotyping occurred in 10 experimental repeats, each lasting 21 days. Seeds were stratified for 4 days and sown on small petri dish plates (12 cm × 12 cm, Novolab) on 1/2 MS medium with 0.6% Gelrite as a gelling agent according to the protocol (Supplementary Word File 1). Plates were placed in the growth chamber, with the following controlled conditions: 21°C and day/night light cycle (16h/8h), under 110 μE/m^2^/s photosynthetically active radiation (cool-white fluorescent tungsten tubes, Osram GmbH, Munich, Germany). After germination, the seedlings of similar size were transferred to large Nunc plates (24 cm × 24 cm) and placed back to the same conditions of growth. Placement of accessions on each plate and placement of plates in the growth chamber were randomized based on incomplete block design. According to this design, each accession was present in each repeat but with a variable number, different for each repeat and each accession. Each plate contained three plants from three different accessions. Col-0 was incorporated in the block design so that it was present in each repeat in a random number. At 14 days after germination, the plates were scanned on a high-resolution flatbed scanner, equipped with backlight illumination (Epson^®^ Expression 11000XL, Seiko Epson Corporation, Nagano, Japan). First, the rosette was excised carefully to keep only the root on the plate for clear imaging. Shoot biomass was measured immediately after excision. The fresh weight of the root was measured instantly after image acquisition.

### Image Analysis and Phenotyping

**FIGURE 1:**
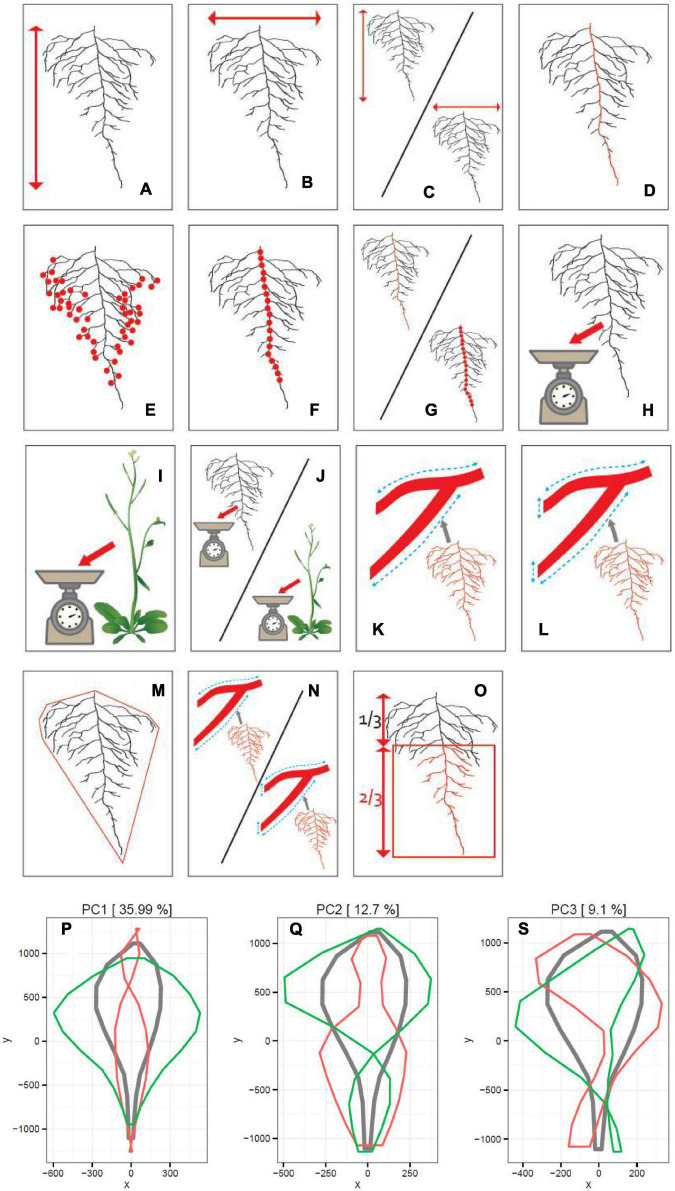
Schematic representation of 15 RSA and 3 PC traits measured on 241 accessions. Panels from **(A–O)** correspond to 15 traits listed in Table 1. In Panels **(P,R,S)**, the results of the PCA are shown. The red line represents the maximum and the green line the minimum values observed. The gray line represents the average of all values for 241 accessions.

Each singular root image has been cut out from a plate picture, with an in-house written script for ImageJ. This program was also used manually to remove artifacts in the root images and maintain one root image per picture. Next, each root picture was analyzed with GiA Roots (Galkovskyi et al., 2012). About 15 traits were analyzed. Six spatial distribution traits have been measured for each image: length distribution, projected area, convex area, solidity, and total root length. Additional two traits, such as the root width and root depth, were analyzed with the use of an in-house written ImageJ script. The total number of LRs and the primary root length were calculated automatically *via* the Root Reader 2D plugin (Clark et al., 2013). The number of first order LRS were scored manually. Subsequently, the root biomass to shoot biomass ratio, width to depth ratio, and LR density were derived from the initial measurements. Additionally, Principal Component Analysis (PCA) was performed with ImageJ plugin routine: MariaJ (Bourne, 2010) on the root shape trait, with the help of the in-house build R script. The shape analysis was obtained by automatically placing pseudo landmarks along with the root profile and extracting their position. The position of the different landmarks was then analyzed using a PCA. This PCA allowed computing the average root system shape and the principal shape modifications observed in the dataset (represented by the different PCs). Altogether, 18 traits have been scored (Figure 1 and Table 1). Correlation coefficients were calculated based on Pearson’s correlation.

**TABLE 1:** Root system architecture (RSA) traits measured on 241 *Arabidopsis thaliana* accessions and broad-sense heritability values (H^2^) measured for 15 RSA traits.

Trait label	RSA trait	Description	Abbreviation	Broad-sense Heritability (H^2^)
A	Root Depth	how deep the root grows	RD	0.4703
B	Root Width	how wide the root grows	RW	0.5412
C	Width to Depth Ratio	ratio of the two values	WDR	0.5347
D	Primary Root Length	length of the main root	PRL	0.4702
E	Total Number of Lateral Roots	amount of all lateral roots emerged	TNLR	0.4001
F	Number of 1st Order Lateral Roots	amount of lateral roots initiation on the primary root	NFLR	0.3322
G	Lateral Root Density	Primary Root Length/Number of 1st Order Lateral Roots	LRD	0.3084
H	Root Biomass	fresh weight of the root	RB	0.2903
I	Shoot Biomass	fresh weight of the shoot	SB	0.3920
J	Root Biomass to Shoot Biomass Ratio	ratio of the two values	RBSBR	0.3738
K	Total Root Length	all lengths of the root system	TRL	0.3474
L	Projected Area	sum whole root system length considering the thickness of root parts	PA	0.4027
M	Convex Area	the area of convex hull that encompasses the root by connecting its most outstanding ends	CA	0.5603
N	Solidity	Projected Area divided by Convex Area	S	0.4961
O	Length Distribution	the fraction of the root length found in lower 2/3 of the whole root system	LD	0.4287
P	Principal Component 1	–	PC1	–
R	Principal Component 2	–	PC2	–
S	Principal Component 3	–	PC3	–

### Rhizotron in Soil Study

To study the RSA in soil, we used an in-house built rhizotron set-up. The rhizotron system consists of flat boxes comprising two plastic sheets with a space of 4 mm filled with potting soil. A detailed protocol on the rhizotron assembly and preparation can be found in the study by Kerstens et al. (2021). The soil substrate in this study was a commercial mix (Aveve potting soil universal use 20L/bag). It was first sieved (Professional sieve; Model: nr 8, total diameter: 450 mm, total height: 100 mm, mesh size: 2.8 mm, diameter: 0.45 mm), dried, and loaded into the rhizotrons in a repeatable manner, allowing for equal density of soil. Next, rhizosheets were submerged into a water bath (2 ml/L Wuxal 8-8-6 solution, diluted with purified water) to ensure saturation of soil in the sheet before sowing. After 24 h, all rhizosheets were placed into scaffolding at an angle of 43 degrees, with the transparent wall facing downward. A micropore tape that was used to seal the top of each sheet during the water saturation period, was removed, and 5 seeds (stratified for 2 days) were placed in the middle of the soil surface. Saran foils were placed for the first 5 days to ensure humidity for germination. After this time, only one well-developed seedling was selected and the others were removed manually. About 5 ml of a diluted (2 ml/L) Wuxal solution^3^ was applied each day to a set of 24 sheets in well-watered conditions. For drought experiments, watering was withdrawn to create mild water and nutrient stress in a parallel set of 24 sheets. Analysis of the root systems was performed 24 days after germination. Plates were scanned on a flatbed scanner (Epson^®^ Expression 11000XL) and additionally, each sheet was photographed including the above-ground parts. Next, the shoots were excised and the length of the inflorescence stems was measured. The rosettes were imaged and the weight of the above-ground parts was determined. The rosette area and the dry and fresh weight of the stem and rosette were measured via an ImageJ in-house created script. The length measurements of roots were scored by hand: Root Width, Depth, PR Length, Convex Area, and Solidity. Additionally, an in-house written ImageJ script was used to determine how root growth is spatially distributed in the sheets. Two models were created. The first model consisted of 4 equal zones out of the whole length of the rhizosheet. The second model used 3 equal zones out of the whole length of the root system. The first model provides insight into the root development in function of the depth of the rhizosheet, while the second is more informative about the overall root development in function of root depth (Supplementary Table 2). Traits measured on both models included centroid, root area distribution, and root distribution.

### Genome-Wide Association Study

Phenotype data were collected for all 15 root traits. Means were calculated for each of the traits, for each accession. Then, broad-sense heritability (H^2^) for all RSA traits was calculated as the ratio of the genetic variation over the total phenotypic variation, with the total phenotypic variation being the sum of the genetic and environmental variation. Genetic variation was estimated as the variance explained by the genotype in a random effect model. The environmental variation was estimated as the residual variation from the random effect model. The random-effect model was constructed using the R package, “sommer” (4). To perform a genome-wide association study (GWAS), a publicly available collection of 250k single nucleotide polymorphism (SNP) array data was used (5). The QTCAT approach was chosen to perform the association analysis based on its in literature highlighted feature to outperform other methods *via* implementation of hierarchical testing to control the population structure (Klasen et al., 2016). As a first step, the means were log-transformed. Subsequently, QTCAT analysis was performed in the R-package program using codes available from the online freeware resource (6) (Supplementary Word File 2). The R analysis automatically records the positioning of significant associations and generates the results as a list and in a visual way as Manhattan plots. Significant SNPs are selected automatically based on family-wise error rate (FWER). Afterward, the genes in the 20kb window around the significant SNP (10 kb up and downward) were searched manually *via* the JBrowse platform (7) and listed (Supplementary Table 5) as genes of interest.

## Results

### Natural Variation of the *Arabidopsis* Taproot System

**FIGURE 2:**
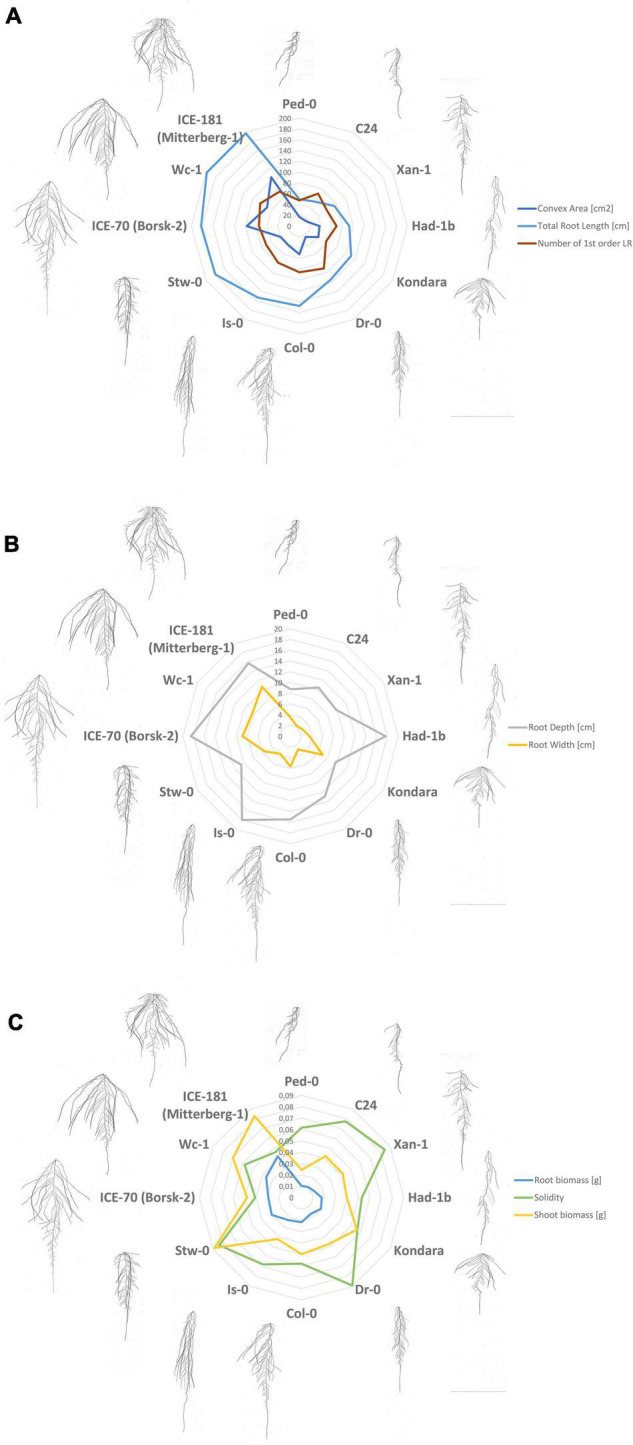
Distribution of RSA traits values for 11 most contrasting accessions and Col-0, represented as scatter plots. **(A)** Convex area [cm^2^], total root length [cm], number of first order lateral roots (LRs). **(B)** Root depth [cm] and root width [cm]. **(C)** root biomass [g], shoot biomass [g], and solidity.

To assess the natural variation of RSA parameters at an advanced stage, 241 *Arabidopsis* accessions were grown in large petri-dishes (24 cm × 24 cm). About 2,659 root pictures were collected and 15 RSA traits were measured (Table 1). Additionally, three traits have been established by means of PCA of root shape. All traits showed a broad variation (Supplementary Figure 1) over the different accessions. The spread of trait values is illustrated in Figure 2, where we selected the 11 contrasting accessions (chosen based on their score as among the 10 highest or lowest values for the following 5 traits: convex area, root depth and width, the total number of LRs, and/or total length) in terms of 9 RSA traits to make scatter plots. The plots directly point not only to accessions that have a similar RSA development but also to some that invest more in one trait over the other. For example, at the level of the convex area, the accessions, ICE-181, WC-1, ICE-70, and Stw-0 contrast with Ped-0, C24, Xan-1, Had-1b, Kondara, and Dr-0 with the first ones showing a much larger convex area (Figure 2A). Furthermore, Ped-0, Xan-1, C24, Kondara, and Stw-0 developed less deep root systems compared to the other accessions (Figure 2B). At the level of solidity, C24, Xan-1, Dr-0, and Stw-O excelled above the others (Figure 2C). All measurements can be found in Supplementary Table 3.

Several traits showed clear correlations (Table 2). Shoot biomass, for example, is strongly positively correlated to root biomass, projected area, and total root length, which confirms that there is an intimate relation between under- and above-ground organs. Other root traits are also positively correlated to shoot biomass, however, in a more moderate manner. The only negative correlation the shoot biomass had was with LR density. Moreover, LR density appeared to be negatively correlated with several other root parameters, such as projected area, solidity, total root length, root biomass, number of first order LR, and the total number of LRs. Root biomass is positively correlated with the convex area, projected area, and total root length but, remarkably as indicated above, not with the lateral root density. This confirms that for an increase in convex area, the elongation of a limited number of root axes rather than the initiation of new ones is important. Overall, the negative correlation of LR density with several parameters suggests that increasing lateral root density may represent an energy-demanding process that occurs at the cost of other growth parameters which might be taken into account in future root-based breeding programs on crops.

**TABLE 2:** Correlation analysis between 15 RSA traits, based on *p*-Values.

	RSA Convex Area	RSA Solidity	RSA Total Length	Shoot biomass	Root biomass	Shoot/Root biomass	RSA Depth	RSA Width	Width/depth ratio	PR length	1st order LRs	Total number of LRs	LR density
RSA Length Distribution	0,256	–0,429			–0,133	0,141		0,301	0,257		–0,225	–0,22	0,368
RSA Area	0,757		0,952	0,725	0,918	–0,374	0,467	0,654	0,466	0,467	0,441	0,339	–0,15
RSA Convex Area		–0,641	0,636	0,444	0,581	–0,248	0,536	0,926	0,701	0,538	0,169		0,235
RSA Solidity					0,146		–0,334	–0,667	–0,546	–0,335	0,185	0,409	–0,503
RSA Total Length				0,718	0,88	–0,327	0,425	0,54	0,371	0,427	0,499	0,402	–0,243
Shoot biomass					0,756	0,234	0,219	0,385	0,304	0,218	0,409	0,387	–0,315
Root biomass						–0,431	0,349	0,487	0,344	0,345	0,412	0,47	–0,216
Shoot/Root biomass							–0,213	–0,199		–0,209		–0,183	
RSA Depth								0,274	–0,148	0,993	0,581		0,144
RSA Width									0,901	0,276			0,251
Width/depth ratio										–0,141	–0,269		0,188
PR length											0,584		0,145
1st order LRs												0,395	–0,696
Total number of LRs													–0,463

*Light green and light red are weak positive and weak negative correlations, respectively. Dark green and dark red shadings represent strong positive and negative correlations, respectively.*

The convex area represents the combination of both the depth and width of the root system. From the correlation data, we could observe that the root width has a stronger positive influence (one of the strongest correlations in the dataset) on shaping the convex area as compared to the root depth of which the positive correlation is rather modest.

Logically, root depth and primary root length are strongly correlated which aligns with a correlation between the convex area and primary root length. Convex area and length distribution were moderately negatively correlated with solidity. Solidity at the same time was strongly negatively correlated with parameters that relate to the width of the root system such as root width and width to depth ratio. In other words, the wider, the more diffuse root systems seem to develop. A similar strong negative correlation was observed between convex area and solidity indicating that reaching a larger area of soil can be accomplished by economizing on root growth in the central part of the root system.

Traits related to LR growth also clearly vary among accessions. Some accessions like ICE-70 and Sap-0, have long laterals that branch out mostly in the shootward region of the primary root and thus are growing in the topsoil levels. In contrast, some accessions, such as Knox-10 or ICE-106 are characterized by shorter but more condensed LRs distributed evenly along the primary root. The total number of LRs was much more variable than the order of LRs. Most of the accessions initiate LRs up to the second order. Only a few reached just the first level of branching. An increase in the number of LRs has a direct impact on how compacted a root system becomes. This relation emerges clearly from the correlation analysis (Table 2). The total number of LRs was positively correlated with solidity and with other traits such as projected area, total root length, shoot biomass, and root biomass.

Additionally, a PCA was used to explain the variation in the shape of the root system between accessions. Since the shape is different from the convex area and is insensible to size, orientation, not rotation, the most appropriate way of scoring the shape of the root system was to give vectoral artificial points to each root system and analyze the PCA. The analysis divided the shape into 3 PCs collectively explaining 58% of the total variation of that trait observed in this dataset. The PC1 was an indicator of the root system width in the upper half of the total root depth, explaining 36% of the whole root shape variation. The PC2 was influenced by the width of the bottom section of the root system, explaining 13% of the variation. The PC1 and PC2 components can be used to discriminate the different genotypes. The PC3 was responsible for about 9% of total shape variation and was indicative of a very peculiar trait representing the bending of the root system (Figures 1O,P,R).

For each accession, the sampling location data is known (longitude and latitude). Based on this data, climate-related parameters (yearly mean temperature and yearly mean rainfall), for each geographical location were assembled. This data collection aimed to analyze if habitat location and/or climate may have any influence on root traits. The influence of habitat conditions on root traits was analyzed as correlations and have also yielded striking dependencies (Table 3). The yearly mean temperature has a moderate negative influence on all analyzed root traits, indicating that higher temperatures might hamper root development. On the other hand, elevation above the sea level is in moderate positive correlation with most of the traits. Yearly precipitation sum has a moderate negative correlation not only with length distribution, convex area, and root biomass to shoot biomass ratio but also has a moderate positive correlation with solidity. This shows that rainfall seems to favor the development of the aboveground parts of the plant. In regions with limited rainfall, root systems appear less compacted thereby increasing the length distribution and area of soil to explore for underground water sources. To further investigate habitat influence on the root traits of the accessions, we have also obtained information about the type of soil occurring in each habitat. Soil types have been converted into numerical values based on the class of soil. Correlation of root traits to the type of soil was, however, not producing significant results, and thus, we could not draw correlations from this analysis (Table 3 and Supplementary Table 4). This might be due to the fact that available taxonomies are not precise for a non-homogenous soil region (mixture of different types soil types).

**TABLE 3:** Correlation analysis between 15 RSA traits and: A - geographical/climate features of their place of origin, B – soil types depending on the soil taxonomy (for the description of soil types, please refer to the Supplementary Table 4).

A	latitude (EW)	longitude (NS)	Country of origin	elevation	Temp Mean {C}	Precipitation sum {mm}	B	soil type 1	soil type 2
RSA Length Distribution				0,147		–0,198	RSA Length Distribution		
RSA Area	0,165	0,236		0,239	–0,349		RSA Area	–0,196	
RSA Convex Area		0,239		0,238	–0,288	–0,128	RSA Convex Area	–0,137	
RSA Solidity						0,162	RSA Solidity		
RSA Total Length	0,19	0,211		0,207	–0,338		RSA Total Length	–0,238	–0,151
Shoot biomass		0,201	–0,163	0,322	–0,187		Shoot biomass		–0,18
Root biomass	0,145	0,205		0,211	–0,296		Root biomass	–0,154	–0,17
Shoot/Root biomass	–0,242				0,222	–0,136	Shoot/Root biomass		
RSA Depth	0,188				–0,152		RSA Depth	–0,181	
RSA Width		0,287		0,3	–0,253		RSA Width		
Width/depth ratio		0,323		0,343	–0,178		Width/depth ratio		
PR length	0,188				–0,153		PR length	–0,186	
1st order LRs	0,149						1st order LRs	–0,16	
Total number of LRs							Total number of LRs	–0,153	–0,162
LR density							LR density		
latitude (EW)				–0,425	–0,565				
longitude (NS)			–0,159	0,253	–0,221	–0,181			
Country of origin						0,139			
elevation					–0,297				
Temp Mean {C}						–0,178			

*Light green and light red shadings are weak positive and weak negative correlations, respectively. Dark green and dark red shadings represent strong positive and negative correlations, respectively.*

### Root System Architecture Identified on Plates Corresponds to Root System Architecture in Soil Conditions

**FIGURE 3:**
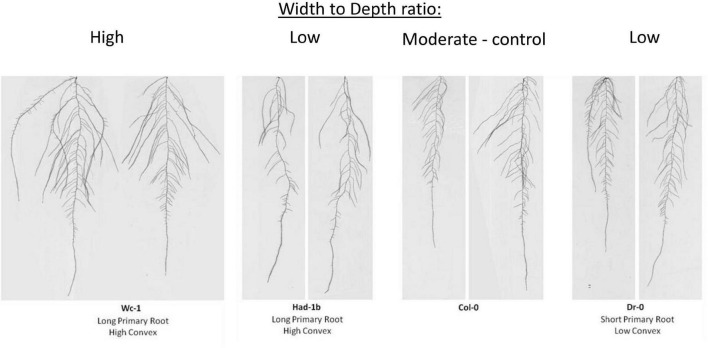
Accessions contrasted width to depth ratio tested in the Rhizotron study. Wc-1 has a low width to depth ratio and a long primary root length and a high convex area. Had-1b has a high width to depth ratio, long primary root length, and a high convex area. Col-0 has an intermediate width to depth ratio, primary root length, and convex area. Dr-0 has a high width to depth ratio but shorter primary root length and a low convex area.

To test whether contrasting phenotypes were stable in the genetically encoded traits or rather the expression of growth under artificial conditions in petri-dishes, we decided to grow a selection of contrasting accessions in soil conditions in a rhizotron set-up. For practical reasons (labor intensity and space limitations to handle the in-house built manual rhizotron system), an experimental setup of 4 accessions was considered feasible. First, we classified the 20 most contrasting accessions based on the width to depth ratio. Following, we narrowed down the selection to 3 accessions, Dr-0, Had-1b, and Wc-1, based on their contrast in primary root length and convex area (Figure 3). Those 3 accessions, and Col-0 as a control, were grown in 6 repetitions in rhizotron boxes (Supplementary Figure 2). Furthermore, we verified the stability of the RSA under high soil moisture content and mild drought conditions (refer to section “Materials and Methods”).

**FIGURE 4:**
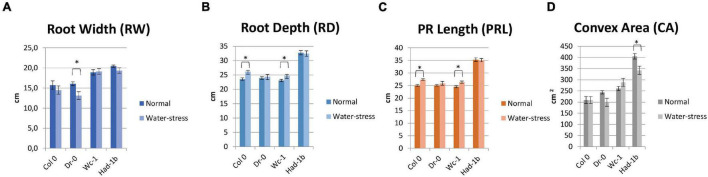
**(A–D)** Results of measurements for root system architecture (RSA) traits, on four accessions tested in the rhizotron set-up, for normal and mild-stress conditions. *, *p* < 0.05 as analyzed by a Student’s *t*-test.

**FIGURE 5:**
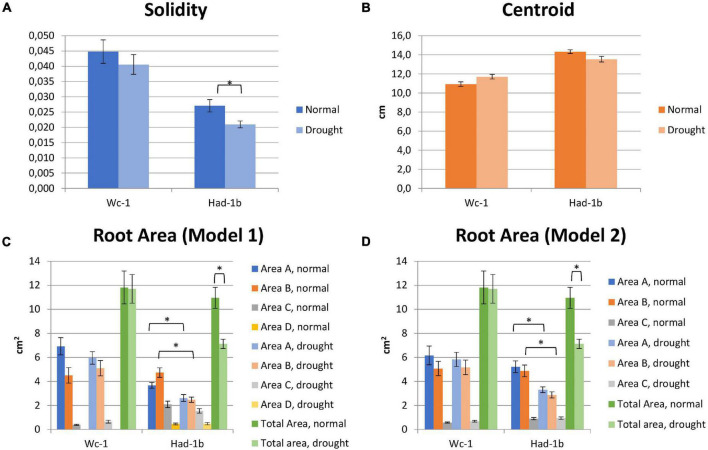
**(A–D)** Results of measurements of RSA traits including root area in the zones of the rhizosheets, for two contrasting accessions. *, *p* < 0.05 as analyzed by a Student’s *t*-test.

**FIGURE 6:**
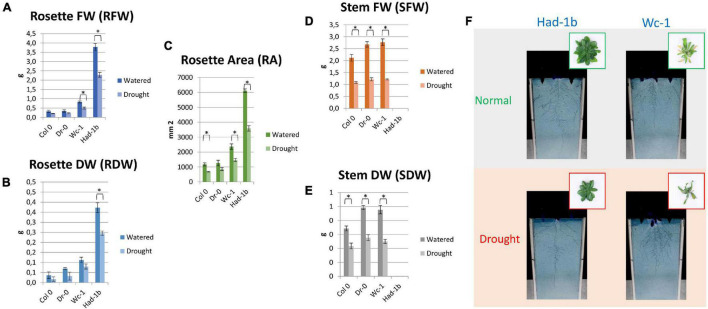
Shoot trait measurements for rhizosheet experiments, for four accessions. **(A–E)** Results of shoot measurements. **(F)** Visualization of shoots of two accessions on 24 DAG in a rhizosheet. *, *p* < 0.05 as analyzed by a Student’s *t*-test.

In conditions of sufficient water availability, Col-0 and Dr-0 performed similarly to each other for all traits measured (Figure 4). For all 4 accessions, root width was similar but PR length and convex area were significantly larger in Had-1b (Figures 4C,D). To analyze how roots are distributed in soil, we have divided the rhizotron sheet surface into zones according to two models (refer to section “Materials and Methods”). The analysis of the distribution of roots in rhizosheets revealed additional characteristics of the Had-1b accession. Had-1b, being the longest accessions from the ones analyzed, represented low solidity indicative of a not densely compacted RSA (Figure 5A). If we look in more detail at the distribution of LRs in different zones, Had-1b had more LRs in the lower zones of the root system, which is represented by the root area in zones. In the case of Model 1 (division into 4 zones), Had-1b had more root area observed in the lower zones of the rhizosheet than Wc-1 (Figure 5C). This is less obvious if the rhizosheet is divided only into 3 zones (model 2) (Figure 5D). Measuring the centroid is an alternative way of getting insight into the spatial distribution of roots. Compared to other accessions, centroid value was higher in the case of Had-1b which seems to be influenced by the production of more LRs in the lower zones of the root system (Figure 5B). This pattern of Had-1b RSA development was observed in well-watered conditions suggesting this accession might be evolutionarily shaped as a water-seeking accession by its deep proliferation of its RSA at the cost of development of LRs and the shoot. Shoot development of plants grown in rhizotron was also scored for the following three parameters: rosette area, rosette dry, and fresh weight; and stem dry and fresh weight (Figure 6). Following its RSA, Had-1b also scored the highest values in the shoot traits. Both, the rosette fresh weight and rosette dry weight were the highest for this accession (Figures 6A,B). On the other hand, this was the only accession that in the period of the experiment did not develop an inflorescence stem (Figure 6D). Its investment into a well-established, deeper root system, prepared for deep water level exploration, influences the rosette area (Figure 6C) but, as a trade-off, it might postpone the progression into the flowering stage (Figures 6D,E). Alternatively, the more extensive root system of Had-1b could be the expression of its prolonged vegetative phase and suggests root traits might follow, in coordination with the shoot, phase-change transitions in plants.

**FIGURE 7:**
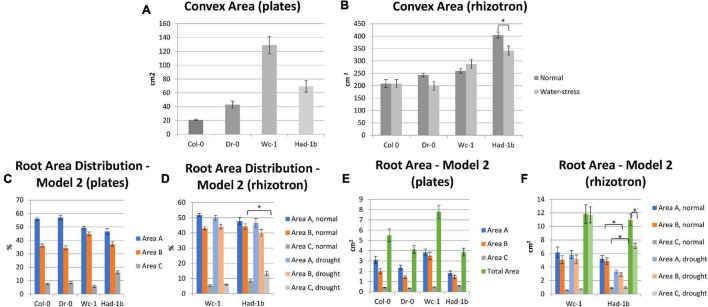
**(A–F)** Comparison between the results of RSA trait measurements in plates (*in vitro*) and in rhizosheets. *, *p* < 0.05 as analyzed by a Student’s *t*-test.

Next, we compared the results obtained in petri-dish conditions with the observations made in the soil (Figure 7). While Had-1b presented the largest convex area in the rhizotrons, in plates it showed an intermediate CA which was much smaller than the one of Wc-1 (Figures 7A,B). Similarly, Had-1b on plates represented a much smaller root area (Figures 7E,F) in comparison with rhizotron conditions, in which it developed equally to Wc-1. Root area distribution for Had-1b and Wc-1, in different zones, was similar on plates as in rhizotrons, indicating that the spatial distribution of the root stays the same over time, only the size can increase depending on growth medium, growth conditions, and stage of development. The different positions of Had-1b relative to the other accessions in soil vs. petri-dishes illustrate that some accessions respond differently and, thus, require more time to reveal their more mature root traits and urge some caution in making conclusions on RSA traits solely based on petri-dish conditions. On the other hand, relative differences of some traits between accessions, such as root distribution between Wc-1 and Had-1b were kept invariant between plates and rhizotron experiments.

### Root System Architecture Parameters Are Influenced by Water Limiting Conditions

Limited water conditions had a visible influence on all accessions. Had-1b still scored the highest values for all parameters in water stress conditions while it showed also a significant reduction in convex area (Figure 4D), solidity, and root area distribution in the upper zones of the root system in comparison to normal conditions (Figures 5A,C). Primary root length did not change in Had-1b under limited water conditions while the other three accessions showed at least a trend in increasing PR length in water stress compared to normal, which was significant for Col-0 and Wc-1 (Figure 4C). On the other hand, Wc-1 did not show a reduction in root width while this was the case for the three other accessions (significant for Dro-0) (Figure 4A).

Concerning the shoot, for all accessions, a drop in values in shoot parameters was observed in water limiting conditions. The size and weight of the rosette were decreased (Figures 6A-C) and the inflorescence stem for the three flowering accessions also developed less as compared to well-watered conditions (Figures 6D,E) clearly showing the negative influence of suspended water treatment on the aboveground traits.

### Genome-Wide Association Studies

**FIGURE 8:**
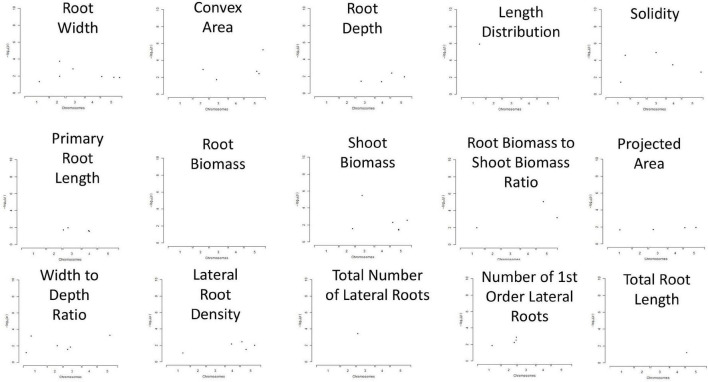
Manhattan plot representation of associated single nucleotide polymorphisms (SNPs) detected for each of 15 RSA traits, plotted on the *x*-axis, based on their genomic position. Loci that are close to each other are averaged as one point in the scheme, for simplified visualization.

For GWAS, a collection of 15 RSA traits was measured on plants grown in *in vitro* conditions, on gel-filled medium plates. The measurements and analysis of traits are described in the section “Materials and Methods.” Broad-sense heritability (H^2^) was calculated and the results varied from 21 to 56% (Table 1). This means that 21–56% of the observed variation for different phenotypes is due to genetic differences. Convex area, root width, and root to width ratio scored above 50% indicating them as the most heritable from our dataset of RSA traits. Root biomass was characterized as the least heritable RSA trait in the dataset. The GWAS analysis was performed with the QTCAT program and indicated 141 significant SNPs for a total of 15 root traits (Table 4 and Supplementary Table 6). The only trait that did not deliver any significant results was root biomass. This is probably linked to low genetically inherited variation in this trait, which scored the lowest for our whole dataset (Table 1). Analysis of a 20 kb window around each significant SNP allowed for collecting a dataset of 412 genes of interest (full list in Supplementary Table 5). The visual representation in the form of simplified Manhattan plots is shown in Figure 8. Some overlap between SNPs was detected for several traits. This was, however, expected due to the natural dependence between root traits. Shared SNPs were detected for the pairs of traits as follows: convex area—projected area, projected area—root biomass, projected area—total root length, root depth—primary root length, root width—convex area (Supplementary Table 6). Those relations are reflected in correlation analysis. A total of 26 genes have been chosen as genes of interest for further analysis (Table 5). The selection of those was narrowed down based on gene ontologies. *The suppressor of MAX 2 1 (SMAX1)* was one of the genes found in the 20 kb window around the SNP associated with shoot to root biomass ratio. The *SMAX1* is known to act downstream of MAX2 and is involved in response to strigolactones and karrikins (Stanga et al., 2016; Moturu et al., 2018). It has a role in germination and seedling morphogenesis but is also expressed in mature roots and axillary shoots. According to recent studies, *SMAX1*, together with the closely related *SMXL2*, plays a role in root and root hair development (Villaécija-Aguilar et al., 2019). In our study, this gene was detected for the shoot to root ratio trait linking the fact that it influences both below- and above-ground plant parts which would fit with the proposed role for strigolactones as coordinators of the shoot and root development. *PIN-LIKE 7 (PILS7)* was also detected for the root-to-shoot trait. It belongs to the known auxin efflux carrier protein family (Rosquete et al., 2018) playing a role in auxin homeostasis which is an essential mechanism for root development. Another gene linked to this analyzed trait is *MILDEW RESISTANCE LOCUS O10*. It is a member of a large family of seven transmembrane domain proteins specific to plants that have a high level of functional redundancy, cofunction, or antagonistic function based on similar or overlapping tissue specificity and analogous responsiveness to external stimuli. It is expressed in root and cotyledon vascular system, in root–shoot junction, and LR primordia, which aligns with the result that was detected for root-to-shoot ratio trait (Chen et al., 2006). From our results, it seems that another resistance gene affects the overall root-to-shoot ratio. *ENHANCED DOWNY MILDEW 1, the* ENHANCER OF TIR1-1 AUXIN RESISTANCE 3 (*SGT1B*) that besides serving a role in plant immunity is also required for the SCF(TIR1)-mediated degradation of Aux/IAA proteins in the canonical auxin signaling pathway, linking it to RSA development (Gray et al., 2003). Two interesting associations for PR length and root depth were identified. One of them, *Root Meristem Growth Factor 6 (RGF6)* or *GOLVEN1*, can also be regarded as a proof of concept of the value of our GWAS as it is required for the maintenance of the root stem cell niche and transit amplifying cell proliferation in the root meristem which would be in agreement with its association with PR length (Ou et al., 2016; Song et al., 2016). The second gene, *C-Terminal Domain Phosphatase-Like 5 (CPL5)*, is involved in the regulation of abscisic acid (ABA) and drought responses of the root and was found in our study to be linked to vertical root growth as well (Jin et al., 2011). Association for convex area yielded a member of a family of LRR receptor-like kinases of which several members were shown to be involved in root growth and development (Ou et al., 2016), namely *RGF1 Insensitive 2 (RGI2)* that also plays role in the maintenance of root stem cell niche by influencing PLETHORA (PLT) transcription factors and is a receptor of RGFs. Association for shoot biomass also emerged as two interesting genes. One candidate gene is *Aberrant Lateral Root Formation 5 (ALF5)* (Diener et al., 2001). The *ALF5* encodes a multidrug efflux transporter gene family member, conferring resistance to toxins. Genes from the same family, *ALF1* and *ALF4* do have mutants with increased levels of endogenous auxin, promoting LR formation by maintaining the pericycle in a mitotically competent state leading to LR formation. ALF4 binds to RBX1 and inhibits the activity of SCFTIR1, an E3 ligase responsible for the degradation of the Aux/IAA transcriptional repressors (Bagchi et al., 2018). Because it was detected for shot biomass trait, it might indicate that influence on those pathways in root development has an influence on the above-ground development. The second gene with the association for shoot biomass is Scare Family Protein 4 (SCAR4) involved in microtubule organization and might again be responsible for shoot biomass increase due to a better root performance, as it was already linked to root elongation (Dyachok et al., 2011). Transcription factors (TFs) are known to take part in the maintenance of auxin signaling in root development. In our candidate gene list, a TF was linked to RSA depth trait: Kow Domain-Containing Transcription Factor 1 (KTF1) which is involved in regulating the transcription by RNA polymerase II (Köllen et al., 2015). Similarly, Basic transcription Factor **3 (BTF3)** was found for width to depth ratio and categorized as a gene of interest for further investigation. Among associations for RSA total length, Dioxygenase for Auxin Oxidation 1 (DAO1) was detected. This is the major indoleacetic acid (IAA) oxidase in plants, manipulating the levels of auxin degradation (Mellor et al., 2016). For this trait also, another oxidase was detected: Auxin Oxidase (DAO2) that is expressed in root caps and is, similarly to DAO1, responsible for auxin homeostasis. Overall, this indicates, as expected, that RSA length is directed by auxin homeostasis mechanisms. Our results of associations lead also to the detection of multiple unknown genes, such as calmodulin-binding protein, described from an auxin-treated complementary DNA (cDNA) library (Reddy et al., 2002). It is detected for the solidity trait, indicating that it might play a role in the initiation of new LRs that increase the compactness of the RSA. The molecular analysis of the genes of interest is beyond the scope of this study and awaits further investigations.

**TABLE 4:** Summary of single nucleotide polymorphisms (SNPs) and genes discovered by QTCAT analysis on 15 RSA traits on 241 *Arabidopsis* accessions.

No.	RSA Trait	QTCAT tool	
		SNPs	Genes
1	Root Depth	11	50
2	Root Width	6	35
3	Width to Depth ratio	28	60
4	Root Biomass	0	0
5	Shoot Biomass	33	36
6	Root Biomass to Shoot Biomass ratio	13	52
7	Projected Area	4	23
8	Convex Area	6	43
9	Solidity	6	45
10	Total Root Length	1	5
11	Length Distribution	4	29
12	Primary Root Length	9	39
13	Total Number of LRs	1	9
14	Number of 1st Order LRs	4	23
15	Lateral Root Density	7	27
TOTAL		141	535
TOTAL (after removing double genes)			412

**TABLE 5:** List of 26 genes selected based on their ontologies as genes of interest, together with information from which traits where those genes detected by QTCAT analysis.

Trait1	Trait2	Gene numbers	Gene name
Convex area		AT5G48940.1	RGF1 INSENSITIVE 2
Root_Shoot_ratio		AT4G11240.1	type I serine/threonine protein phosphatase
Root_Shoot_ratio		AT4G11260.1	ENHANCED DOWNY MILDEW 1, ENHANCER OF TIR1-1 AUXIN RESISTANCE 3
Root_Shoot_ratio		AT4G19120.1	EARLY-RESPONSIVE TO DEHYDRATION 3
Root_Shoot_ratio		AT4G20140.1	GASSHO1, GSO1, SCHENGEN 3, SGN3
RSA_depth		AT1G47056.1	VIER F-BOX PROTEINE 1
RSA_depth	PR_length	AT3G19600.1	C-TERMINAL DOMAIN PHOSPHATASE-LIKE 5
RSA_depth	PR_length	AT4G16515.1	ROOT MERISTEM GROWTH FACTOR 6
RSA_depth		AT5G04290.1	KOW DOMAIN-CONTAINING TRANSCRIPTION FACTOR 1
RSA_length		AT1G14120.1	ATDAO2, AUXIN OXIDASE
RSA_length		AT1G14130.1	DIOXYGENASE FOR AUXIN OXIDATION 1
RSA_length		AT1G62810.1	COPPER AMINE OXIDASE1
Shoot_Biomass		AT3G23560.1	ABERRANT LATERAL ROOT FORMATION 5
Shoot_Biomass		AT5G01730.1	SCAR FAMILY PROTEIN 4, WAVE3
Shoot_Root_ratio		AT5G57710.1	SUPPRESSOR OF MAX2 1
Shoot_Root_ratio	Root_Shoot_ratio	AT5G65970.1	MILDEW RESISTANCE LOCUS O 10
Shoot_Root_ratio	Root_Shoot_ratio	AT5G65980.1	PIN-LIKES 7
Solidity		AT5G40190.1	calmodulin-binding protein
Width_Depth_ratio		AT1G17880.1	BASIC TRANSCRIPTION FACTOR 3
Width_Depth_ratio		AT3G16690.1	SWEET16, Nodulin MtN3 family protein
Width_Depth_ratio		AT3G16785.1	PHOSPHOLIPASE D P1
1st order LRs		AT2G46920.1	Protein phosphatase 2C family protein
LR Density		AT4G13930.1	SHM4, serine hydroxymethyltransferase 4
RSA_depth		AT1G47128.1	RESPONSIVE TO DEHYDRATION 21
Root_Shoot_ratio		AT5G59240.1	Ribosomal protein S8e family protein
Root_Shoot_ratio		AT5G59290.2	UDP-GLUCURONIC ACID DECARBOXYLASE 3

## Discussion

### Root System Architecture Phenotyping: A Gateway to Underground Variability

With the current state of phenotyping of underground traits, common techniques can be upgraded and manipulated to fit more precisely to the investigation of desired root traits. In our case study, the exchange of a gelling agent from agar to Gelrite allowed for a limitation of root waving, due to its harder for a root to penetrate properties (Yan et al., 2017). This allowed us to obtain a less tangled RSA in more mature root systems and enabled the extraction of a long list of root traits, that can precisely describe RSA on a miniaturized scale. LR traits can be measured without having to sacrifice plants. Usually, root tracing in *in vitro* experiments is performed on very young seedlings compared to our study. At the end of the day, the phenotyping method, however, will always be a limiting factor as there is no single, universal method that allows for investigating all possible root traits at once (Deja-Muylle et al., 2021). The possibility to combine phenotyping techniques, despite time and cost consuming, allows for the extraction of additional information. In our case, a rhizotron study gave us information about differences between *in vitro* studies and soil growth on a selected group of accessions.

A high degree of RSA variability exists in the pool of the analyzed *Arabidopsis* accessions. Similar results of the high variability of RSA traits were described in a recent *Arabidopsis* root study in the “GLORoots” rhizo-systems (Larue et al., 2021). We have confirmed a strong relationship between the above and underground plant traits. An increase in shoot biomass had only one negative correlation to LR density, indicating that the overall increase in area that the root systems take, is due to a shift from the elongation of the primary root to the initiation of a new first order LRs. The LRs have an influence on total root length increasing the soil exploration zone for nutrients and thus influencing the shoot biomass as well. LR density is overall a trait that negatively correlates with many other measured traits, suggesting that LR density is a high energy demanding process, that occurs at a cost of other parameters. Overall, the LR density and dependencies with other traits should be considered while selecting root traits in breeding programs. Understanding the correlations between RSA traits and between under and aboveground parts should be considered as one of the best approaches for plant improvement (Paez-Garcia et al., 2015), especially as it can be linked to certain root ideotypes that aim at the improvement of nutrient and water uptake, leading to higher yield (Lynch, 2019).

### Rhizosheets vs. Petri-Dishes and Water Deficit Stress

Recently, the study of the hidden half is gaining increased interest among researchers, resulting in various attempts to phenotype the root systems of plants. However, many of the developed techniques are destructive or inaccurate when being deployed in the field or being relatively expensive to install and space-consuming (Atkinson et al., 2019). Techniques also depend on which plant species is under evaluation (Deja-Muylle et al., 2021). Our aim was not only to test RSA observed on plates but also to provide a low-cost yet versatile root phenotyping system without the immediate requirement for sophisticated equipment allowing for easy implementation in any laboratory. Our experiments confirmed that ecotypes of *A. thaliana*, distinguished by specific root traits observed on gel media in *in vitro* studies, maintained their phenotypes in soil, but not for all four accessions. This means that there is a certain overlap between traits in *in vitro* studies at the small, young seedlings and later stages of plant development. This seems, however, to be sensitive to accession genetic build-up as, shown on accession Had-1b. Its differential performance between two phenotyping systems might be explained by the fact that for some accessions, certain root traits become more pronounced in the later stages of development. In such cases, the rhizotron has more benefits over *in vitro* techniques in plates as it allows for the plants to grow bigger, with fewer constraints of the physical system in which there are grown. Additionally, different phenotyping techniques bring versatile interference obstacles that cause variation in RSA. Possibly, in certain accessions, mechanically responsive genes might become activated only in defined conditions due to increased friction of soil medium compared to gelling agent, leading to differences in spatial distribution. Such a situation has been reported by Zhao et al. (2017), in experiments performed *in vitro* and in sand-filled rhizotrons on pea genotypes. Our results confirm the same result that seedling root traits are not *per se* predictive of mature RSA over time. Moreover, the plasticity in RSA is specific for each accession and, thus, represents a very interesting resource to study further to be used in crop breeding aiming at the production of crops that efficiently adapt to changing environments (Schneider and Lynch, 2020; Van Der Bom et al., 2020).

Rhizotron systems have also brought a valuable approach to test if and how RSA changes upon applied stress. We have tested a mild water stress setup on three contrasting accessions. Water stress did influence all RSA types tested. Among those accessions, there was one, Had-1b that seemed to be, however, less affected by water limitation than others, resulting in a more limited decrease in the observed root traits. The result of this experiment is that traits observed in *in vitro* conditions are not predictive of how plants will react to water stress. Had-1b and Wc-1 both had one trait that was not changed under water-limitation compared to the other two accessions. This result is corroborated by the study of Guimarães et al. (2020), based on a study of rice japonica accessions, which demonstrated that there was no indication of RSA traits on how plants, even the ones representing the same values of traits, react to water stress.

### The Power of Genome-Wide Association Studies to Detect Genes Involved in the Overall Root System Architecture Development

In our study, we have detected a high amount of genes of interest, similar to many other root GWASs (Deja-Muylle et al., 2021). This might be explained partially due to the nature of the QTCAT program that was chosen for this analysis. It outperforms other algorithms, by specific correction of population structure leading to the identification of more loci while at the same time minimizing the amount of false-positive errors (Klasen et al., 2016). In this way, it efficiently counterparts the drawbacks that GWAS analysis struggles with currently (Alseekh et al., 2021). Another reason behind the high amount of SNPs detected is due to the intricate nature of RSA traits that are often intertwined and redundant, causing many genes to be responsible for one root trait (Motte et al., 2019). To narrow down the number of candidate genes, we have employed our prior knowledge over biological pathways and known gene ontologies, which is a common way of limiting GWAS results. In the case of this study, 26 genes have been selected and, similarly to many other studies in *Arabidopsis* roots, further work is necessary, to uncover the pathways and molecular role in driving the RSA trait development of those genes (Kawa et al., 2016; Ristova et al., 2018). We have detected a few genes with known functions in the root development that are a good proof of concept for the chosen GWAS method. Additionally, the fact that some genes known to be involved in certain root development pathways have been identified in our study as responsible for another, distinct root trait, indicating that those will be an interesting subject in the further analysis as many genes are known to participate in multiple pathways. We have detected genes involved in different processes, for example, in hormonal signaling such as auxin, ABA, and strigolactones. Few of the indicated genes were linked to root–shoot interactions. We also retrieved genes involved in drought responses or genes that have roles in germination or disease resistance. This wide span can be indicative of GWAS detecting multiple casual variants. New methods are developed to further fine-tune this type of result (LaPierre et al., 2021). Based on our results, we can conclude that RSA is an interesting topic to study by means of GWAS, as it can reveal genes that would have not been linked to specific RSA traits by other means, such as forward genetics. There are two recent examples where GWAS appeared to be successful in pinpointing genes responsible for RSA expansion (Ogura et al., 2019; Waidmann et al., 2019). Additionally, a successful GWAS was performed on RSA traits measured in a rhizotron system called GLO-ROOTS, where roots are traced after fluorescent labeling of accessions by introducing a genetically encoded reporter system (Larue et al., 2021). This is because GWAS on RSA fulfills all requirements for a successful GWAS setup. With the correct phenotyping setup, a vast number of root traits can be mined. Second, the correct sample size will lead to increased GWAS power by saturating the dataset with the variability in the trait of interest. Based on our phenotyping experiment, it is clear that high variability in the root traits among the accessions for *Arabidopsis* is present. A trait with the lowest heritability (root biomass) yielded no significant SNPs, confirming that low heritability escapes the power of GWAS to detect causative regions of the genome.

Indeed, the GWAS produces a high amount of data that can be further exploited. For instance, in crops, marker-trait associations are used for breeding strategies (Gupta et al., 2020). Our resulting list of genes of interest is long and even after selection, it still requires molecular validation, which becomes a time-consuming drawback. It is, however, important to note that the GWAS technique, not only indicates association to the genes with mutations in gene expression but also can detect, for example, regions responsible for protein structure or post-transcriptional modifications. This opens up an excellent way of looking for gene-phenotype relationships and should be used in root studies to help undercover more of the still unknown gene pathways. Furthermore, the GWAS analysis is evolving yearly with new algorithms, techniques, and databases available (Togninalli et al., 2018). The phenotype data collected in this study is not of one-time use as it can, in the future, be implemented as a source for more advanced GWAS gene detection or as a part of metanalysis to link different GWAS studies in *Arabidopsis* enhancing our insight into genotype-phenotype relationships.

## Data Availability Statement

The datasets presented in this study can be found in online repositories. The names of the repository/repositories and accession number(s) can be found in the article/Supplementary Material.

## Author Contributions

AD-M and TB conceptualized the manuscript. AD-M performed the phenotyping and GWAS experiments. AD-M, BP, HM, and TB wrote the manuscript. AD-M and DO performed the rhizotron experiments. AD-M and MN made the figures. VS, GL, and PC shared their GWAS expertise and plant material. All authors contributed to the article.

## Conflict of Interest

The authors declare that the research was conducted in the absence of any commercial or financial relationships that could be construed as a potential conflict of interest.

## Publisher’s Note

All claims expressed in this article are solely those of the authors and do not necessarily represent those of their affiliated organizations, or those of the publisher, the editors and the reviewers. Any product that may be evaluated in this article, or claim that may be made by its manufacturer, is not guaranteed or endorsed by the publisher.

## Footnotes

1
https://climatecharts.net/

2
https://data.isric.org/geonetwork/srv/pol/catalog.search#/home

3
https://www.certiseurope.be/producten/productwijzer/productpage/product/wuxal-super-8-8-6/#skip-content

## Supplementary Material

Supplementary Figure 1 Natural distribution of RSA values measured on 241 accessions illustrated for 4 root traits, for example, **(A)** Length distribution, **(B)** solidity, **(C)** total root length. The position for Col-0 is indicated in red. X scale is in [cm] for TRL and in [cm^2^] for Convex Area.

Supplementary Figure 2 Four accessions grown in rhizosheets at 24 DAG (W-normal water conditions, D: mild drought conditions).
