# Supplementary figures and images for: Genetic Variability of Arabidopsis thaliana Mature Root System Architecture and Genome-Wide Association Study
Source: Front Plant Sci. 2022 Jan 28;12:814110. doi: 10.3389/fpls.2021.814110 (PMC8831901; doi:10.3389/fpls.2021.814110)

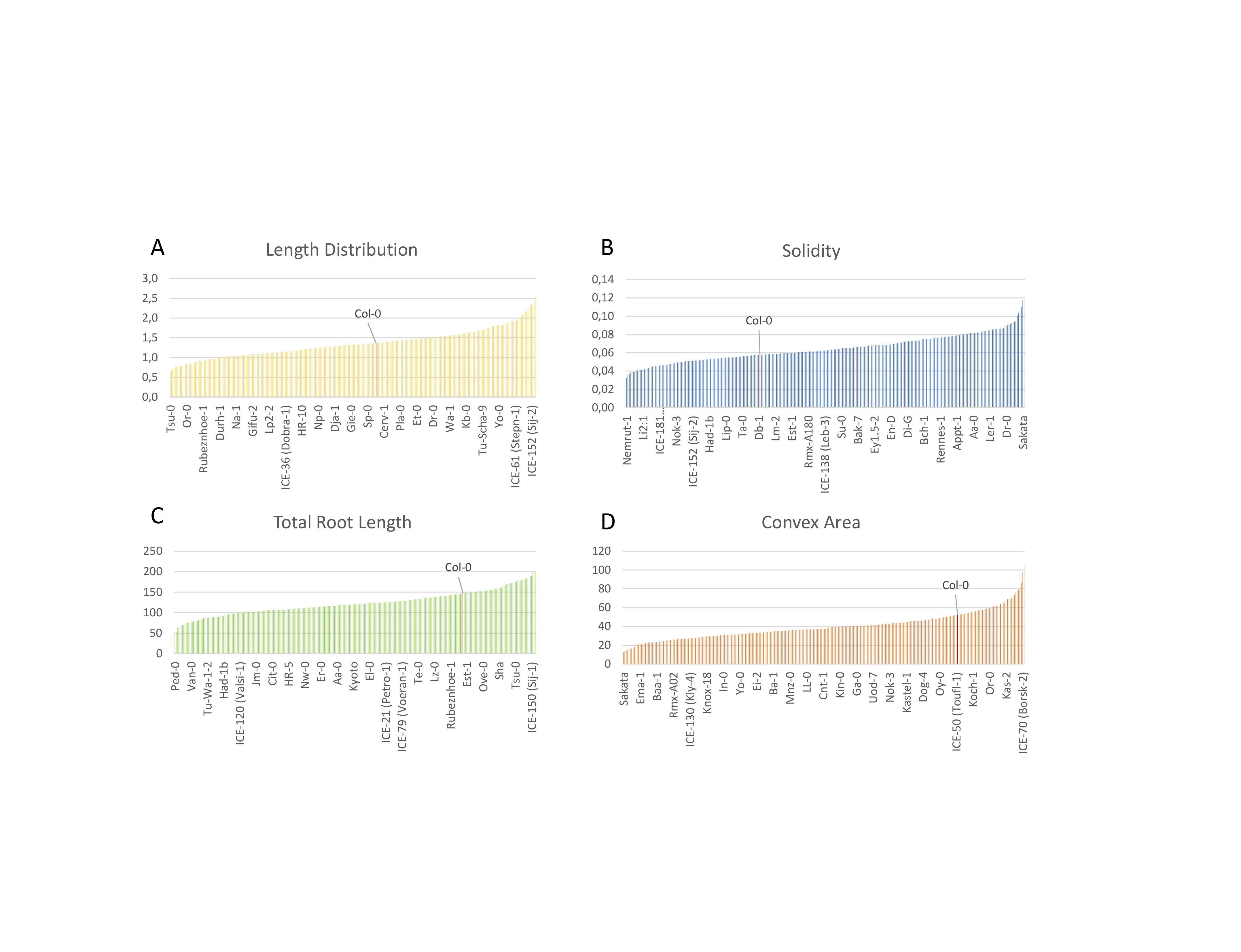

Supplement: Supplementary Figure 1 — Natural distribution of RSA values measured on 241 accessions illustrated for 4 root traits, for example, (A) Length distribution, (B) solidity, (C) total root length. The position for Col-0 is indicated in red. X scale is in [cm] for TRL and in [cm2] for Convex Area. [file Image_1.JPEG]

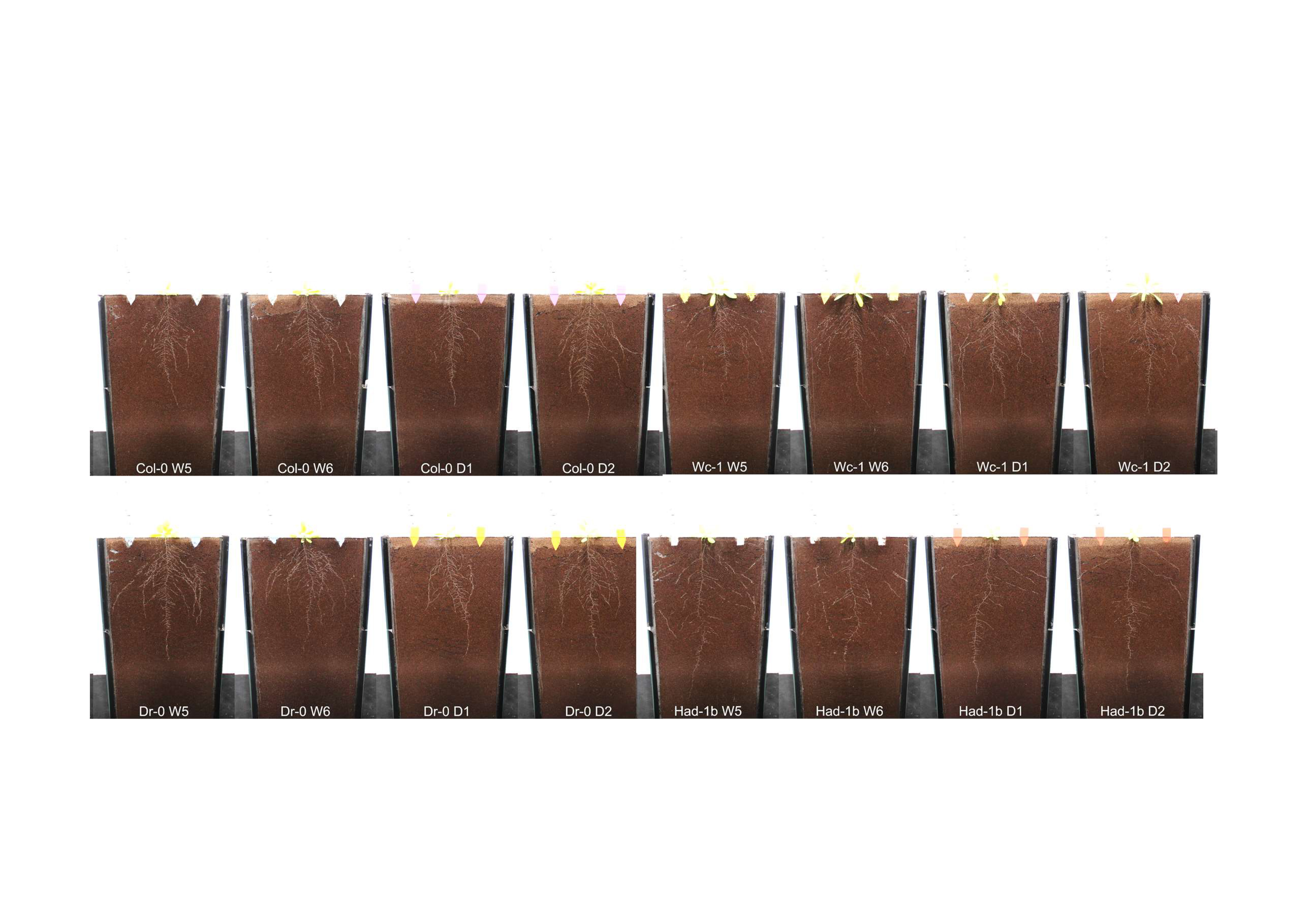

Supplement: Supplementary Figure 2 — Four accessions grown in rhizosheets at 24 DAG (W-normal water conditions, D: mild drought conditions). [file Image_2.JPEG]
