# Supplementary material for: Genetic Variability of Arabidopsis thaliana Mature Root System Architecture and Genome-Wide Association Study
Source: Front Plant Sci. 2022 Jan 28;12:814110. doi: 10.3389/fpls.2021.814110 (PMC8831901; doi:10.3389/fpls.2021.814110)
Supplement: Supplementary file 10 [file Table_7.DOCX]

Medium preparation for phenotyping of Arabidopsis accessions:

- Prepare medium for 1L:

½ MS – 2,154 g

Myo-inositol – 0,1g

MES buffer – 0,5g

Sucrose – 10g

ddH_2_O fill up to 1L

- Set the pH to 5,7

Add gelrite – 6g/1L

- Set for autoclaving (afterwards store in 60 degrees – gelrite can’t be REMELTED!)
- Pour under the flow into plates. (200 ml per plate for large plates, 50 ml per plate for small plates)
- Leave to solidify for about half an hour.
- Sow plants

Medium preparation for phenotyping of Arabidopsis accessions:

- Prepare medium for 1L:

½ MS – 2,154 g

Myo-inositol – 0,1g

MES buffer – 0,5g

Sucrose – 10g

ddH_2_O fill up to 1L

- Set the pH to 5,7

Add gelrite – 6g/1L

- Set for autoclaving (afterwards store in 60 degrees – gelrite can’t be REMELTED!)
- Pour under the flow into plates. (200 ml per plate for large plates, 50 ml per plate for small plates)
- Leave to solidify for about half an hour.
- Sow plants

Protocol for Rhizotron preparation and seeds sowing:

**Sieving of soil**

Apply some soil on the sieve with basket underneath to catch the substrate. Apply little force while swiping the soil across the mesh of the sieve. The crumbs and residue left is discarded into the soilbag, the sieved soil is being sieved once more via the same process. (When pausing the preparation make sure to cover the sieved soil to protect it from drying, also make sure the original soil bag is closed in between the sieving process)

**Filling of the rhizosheets**

Fill the rhizosheet all the way to the top When the rhizotron is filled completely, a downward force is applied in order to shake the soil and compact it.

- Fill to top, compact to 1/2 the height of the rhizosheet
- Fill to top, compact to 2/3 the height of the rhizosheet
- Fill to top, compact to 3/4 the height of the rhizosheet
- Fill to top, compact to 7/8 the height of the rhizosheet
- Fill to top, compact to 15/16 the height of the rhizosheet

As last step the rhizosheet is filled to the top. Next soil is to be put onto the top of the rhizosheet, after which force is applied to push it in the gap, this step is repeated afterwards two times. To seal, a layer of Micropore tape is applied at the top in addition.

**Waterbath**

The soil needs to be saturated with liquid. This is done by submerging the sheets in a waterbath.

**Assembly of the rhizobox**

The sheets are placed at 43 ° in the rhizobox, avoid putting the sheets in a straight position, as this can cause collapse of the layer of soil while excess of water is leaking out.

**Rhizotron maintenance**

The saran foil is removed 6 days after sowing and one seedling is selected to continue growth on the rhizosheet. From that day onward, 5 ml of the diluted wuxal solution is applied each day (make sure the prepared solution is covered from light). The holes generated by the 2 plastic pieces put in earlier allow 2.5 ml at each plastic piece. In this manner Arabidopsis plants can easily be grown for a month before final acquisition and/or harvest is required.
