# Supplementary material for: Genetic Variability of Arabidopsis thaliana Mature Root System Architecture and Genome-Wide Association Study
Source: Front Plant Sci. 2022 Jan 28;12:814110. doi: 10.3389/fpls.2021.814110 (PMC8831901; doi:10.3389/fpls.2021.814110)
Supplement: Supplementary file 9 [file Data_Sheet_1.docx]

R code used to perform QTCAT

```
# loading QTCAT package

    library (qtcat)

    require(qtcat)

# loading of phenotype data (pdata) and SNP positions (snp)

    pdat <- read.csv2("file title with phenotype data per trait", header = TRU

    snp <- read.snpData("SNP_data_from_1001_Genomes.csv", sep = ",")

# clustering of SNP positions

    clust <- qtcatClust(snp)

# constructing a S3-object containing a SNP design matrix and a hierarchy

    geno <- qtcatGeno(snp, clust)

# constructing a S3-object containing phenotype and if additional covariats matrix of those

    pheno <- qtcatPheno(names = pdat[, 1],

        pheno = pdat[, 2]
```
